# Follicle Stimulating Hormone Receptor (FSHR) Polymorphisms and Polycystic Ovary Syndrome (PCOS)

**DOI:** 10.3389/fendo.2019.00023

**Published:** 2019-02-12

**Authors:** Joop S. E. Laven

**Affiliations:** Division of Reproductive Endocrinology and Infertility, Department of Obstetrics and Gynaecology, Erasmus University Medical Center, Rotterdam, Netherlands

**Keywords:** FSH, LH, testosterone, polymorphisms, PCOS

## Abstract

Polycystic ovary syndrome (PCOS) is the commonest endocrine abnormality in women of reproductive age typically presenting with chronic oligo- or anovulation, clinical, or biochemical hyperandrogenism and polycystic ovarian morphology (PCOM). Restoring mono-ovulation is the ultimate goal of ovulation induction and most women do respond to ovulation inducing agents causing their Follicle-stimulating hormone (FSH) levels to rise. Familial clustering and the results from twin studies strongly support an underlying genetic basis for PCOS. Recent Genome wide association studies (GWAS) have identified several genetic variants being genome wide significantly associated with PCOS. Amongst those are variants in or near the Luteinizing hormone (LH) and FSH receptor genes as well as a variant in the FSH-β gene. The aim of this review is to summarize the available evidence as to whether single nucleotide polymorphisms are able to modify the PCOS phenotype or whether they constitute a risk factor for the syndrome. Data on the role of FSHR polymorphisms in PCOS are conflicting. It seems that in large Chinese studies FSHR polymorphisms are not associated with either PCOS risk or with PCOS treatment outcome. However, in large scale studies in Caucasians these polymorphisms seem to influence the risk of having PCOS. Moreover, these studies also showed that some polymorphisms might affect some clinical features of PCOS as well as treatment outcome. Although most research has focussed on the role of FSHR polymorphisms there seems to be also some evidence showing that single nucleotide polymorphisms (SNPs) in the LHCG-Receptor as well as those in FSH-β gene might also alter the phenotype of PCOS. In conclusion most studies confirm that FSHR polymorphisms do alter the phenotype of PCOS in that they either alter the response to exogenous FSH or hat they increase the risk of having PCOS.

## Introduction

Polycystic ovary syndrome (PCOS) is the commonest endocrine abnormality in women of reproductive age, estimated to affect 5–20% of women in most populations (1). It is a heterogenous syndrome and according to the Rotterdam Criteria, PCOS is recognized in women who have two of three symptoms: chronic amenorrhea, clinical, or biochemical hyperandrogenism and the ovarian morphology of polycystic ovarian morphology (PCOM) in ultrasound imaging, after other reasons have been excluded (2). The syndrome is also associated with distressing cutaneous manifestations of androgen excess such as hirsutism and acne (3).

The typical biochemical features are elevated serum concentrations of testosterone and luteinizing hormone (LH), but PCOS is also associated with a characteristic metabolic disturbance that includes insulin resistance, hyperinsulinaemia, and abnormalities of energy expenditure. Crucially, PCOS is now recognized as a major risk factor for the development of type 2 diabetes (T2D) and cardiovascular disease in later life. Women with PCOS have a 3 to 7-fold increase in risk of T2D (3). At least in part, this reflects the strong associations between PCOS and obesity, with the latter a frequent concomitant, and likely amplifier, of the PCOS state (4).

Finally, it represents the major cause of anovulatory infertility, involving up to 20% of infertile couples. Restoring mono-ovulation is the ultimate goal of ovulation induction strategies. Typically these women do respond to ovulation inducing agents causing their own endogenous follicle-stimulating hormone (FSH) levels to rise and initiate ovulation or inducing the latter by administering exogenous FSH to them (5).

## Genetics of PCOS

PCOS clusters within families and having a first degree relative suffering from PCOS conveys a 25% risk of either developing the full blown clinical picture or having a 25% risk of sharing characteristics of the syndrome amongst siblings to other siblings (6, 7). Similar results were obtained in several twin studies showing higher rates of concordance in PCOS characteristics between monozygotic twin sisters compared to dizygotic twins (8). Taken together these data strongly suggest a complex genetic basis for PCOS.

Several studies have attempted to explain the high overall prevalence of PCOS among women worldwide despite its link to subfertility and thus constituting an evolutionary paradox. Recently it has been shown that several genetic loci associated with the disease differently modulate the reproductive parameters of men and women. This observation suggests that these genetic variants lead to opposite effects on reproductive success in women and men. Intra-locus sexual conflict as a cause of the persistence of PCOS supports the high prevalence throughout evolution in humans (9).

To date, large numbers of genetic studies have identified over 200 susceptibility genes that might be functionally related to PCOS. However, the vast majority of these have not been replicated in other studies (10). Because PCOS seems to be a complex multigenic disease a more comprehensive, unbiased, non-hypothesis driven approach seems to be more informative. Hence high throughput genome association studies (GWAS) should be performed in order to unravel the genetic background of PCOS.

**Figure 1:**
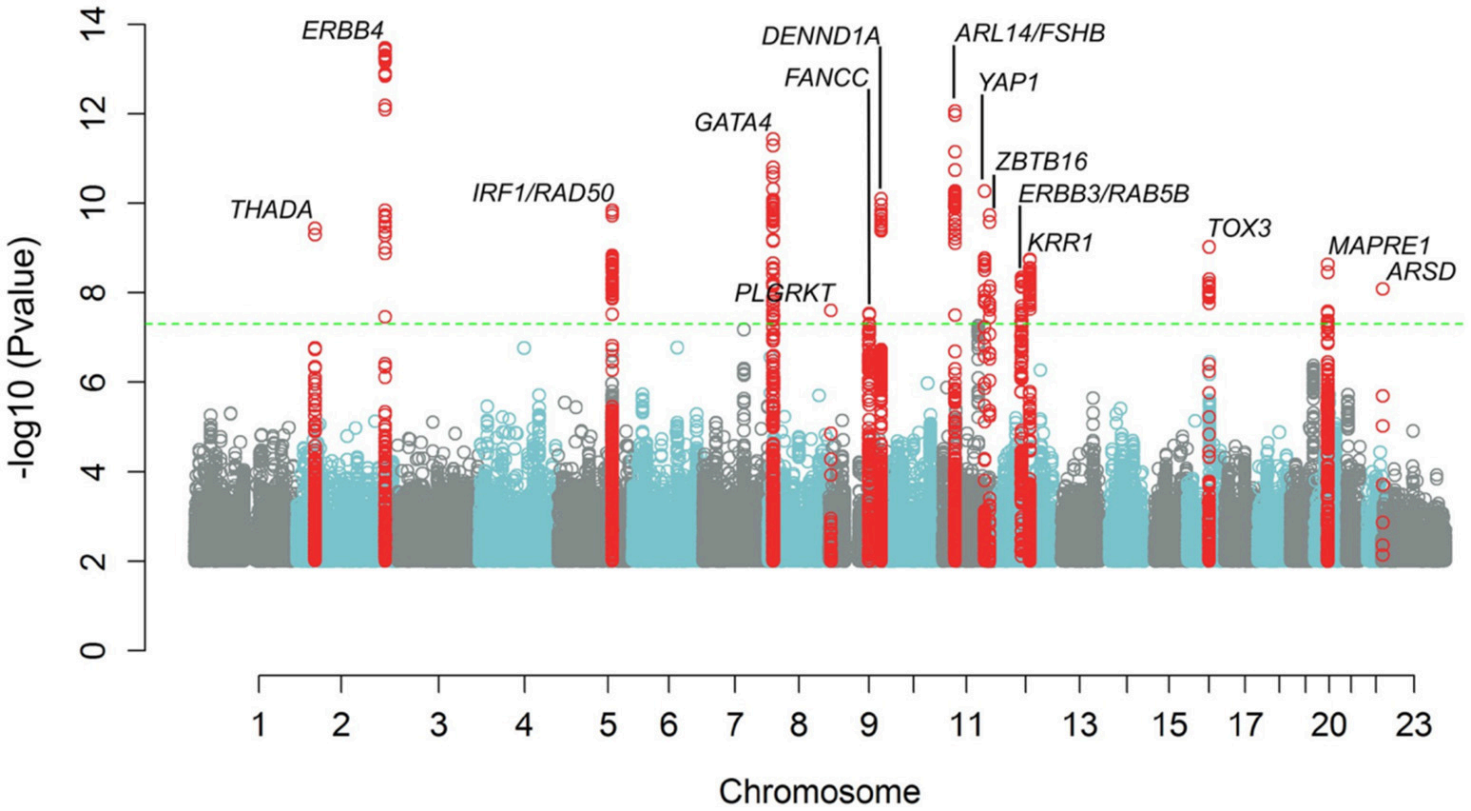
Manhattan plot showing results of meta-analysis for PCOS status, adjusting for age. The inverse log10 of the *p* value (−log10(*p*)) is plotted on the Y axis. The green dashed line designates the minimum *p* value for genome-wide significance (<5.0 × 10^−8^). Genome wide significant loci are denoted with a label showing the nearest gene to the index SNP at each locus. SNPs with *p* values <1.0 × 10^−2^ are not depicted.

Recent GWAS have identified up to 18 genetic variants being genome wide significantly associated with PCOS (11–14) (Figure 1). Amongst those are variants in or near the LH (LHCGR) and FSH receptor (FSHR) genes as well as a variant in the FSH-β gene. Most of the identified genes have been identified and replicated in Chinese, South-East Asians as well as in Caucasian populations (15). Moreover, Mendelian randomization analyses indicate causal roles in PCOS etiology for higher body mass index (BMI), higher insulin resistance, and lower serum sex hormone binding globulin concentrations. Furthermore, genetic susceptibility to later menopause is associated with higher PCOS risk and PCOS-susceptibility alleles are associated with higher serum anti-Müllerian hormone concentrations in girls (14). In a recent paper the functional roles of strong PCOS candidate loci focusing on FSHR, LHCGR, insulin receptor (INSR), and the DENND1A gene were reviewed. The authors of this paper propose that these candidates comprise a hierarchical signaling network by which DENN domain containing 1A (DENND1A), LHCGR, INSR, RAS oncogene family member 5B (RAB5B), adapter proteins, and associated downstream signaling cascades converge to regulate theca cell androgen biosynthesis (16).

## FSHR Polymorphisms

The FSHR gene is located on chromosome 2 p21-p16 and consists of 10 exons and 9 introns. The first 9 exons encode for the extracellular domain of the receptor, whereas exon 10 encodes for the C-terminal end of the extracellular domain, the entire transmembrane domain and the intracellular domain of the FSHR. Exon 10 is fundamental for signal transduction, but it is not necessary for ligand binding. Up till now around 1,800 SNPs of the FSHR gene have been reported in the National Centre for Biotechnology Information (NCBI) SNP database (http://www.ncbi.nlm.nih.gov). SNPs are located either in the coding regions (exons, 8 SNPs), or within intronic regions of exons. Only 1 SNP is located in the 5′ untranslated region of the FSHR mRNA position−29 (ss2189241). Of the eight SNPs within coding regions 7 are located in exon 10 at codon positions 307, 329, 449, 524, 567, 665, and 680. Six of the latter SNPs eventually result in amino acid substitution and are therefore non-synonymous. The two best characterized polymorphisms as far as their allele frequencies and ethnic distribution is concerned are the Ala307Thr (rs6165) and the Ser680Asn (rs6166). Both of these polymorphisms are linked to each other during recombination in a way that they always occur together (17).

Several studies aimed to correlate the FSHR polymorphism and ovarian function. The polymorphism at position 680 harboring Serine residues at both alleles is associated with higher endogenous FSH serum concentrations and a longer follicular phase length. This suggest that this FSHR variant is less sensitive to FSH. Indeed, women having the Ser/Ser variant needed more exogenous FSH during their ovarian stimulation phase during IVF treatment cycles. Moreover, Asn680Ser polymorphism was not associated with premature ovarian failure (POI). Finally, in women with PCOM the distribution of the two allelic variants greatly varied amongst different studies (17–20).

## FSH Receptor Polymorphisms and PCOS

### FSHR Polymorphisms and PCOS Susceptibility

The first study ever which screened the entire coding region of FSHR gene for pathogenic mutations in 124 patients with PCOS and found no mutations in these patients. The two well-known polymorphisms, Thr307Ala and Ser680Asn showed similar distributions of the allelic variations and protein isoforms in PCOS compared to 236 normal healthy control subjects. It appears from this study that mutations in the coding regions of FSHR gene are not a causative factor for PCOS in Chinese Singapore women (21). Similarly, Fu and co-workers recruited 384 unrelated PCOS patients and 768 healthy individuals from the Shaanxi province in the northern part of China. The Ala307Thr and Ser680Asn polymorphisms were studied together with clinical characteristics of the study subjects in a case-control setting. The frequency of FSHR Ala307Thr and Ser680Asn variants along with the haplotype were not significantly different between the PCOS patients and the controls although the Ser680 variants seemed to be associated with higher levels of endogenous FSH and low estradiol levels. This study suggests that the two variants of the FSHR gene are not a causative factor of PCOS in Northern Chinese Han women (22). In another study in Han Chinese women suffering either from PCOS (*n* = 215) or being healthy controls (*n* = 205) recruited from Shanxi Province in north China the Ala307Thr and Ser680Asn polymorphisms of FSHR were not associated with PCOS. However, the FSHR polymorphisms were related to the endogenous serum concentrations of FSH and Prolactin. No other PCOS-associated endocrine hormones as well as clinical pregnancy rates in PCOS patients were recorded in that study (23). Only one study in Chinese women revealed a significant between FSHR polymorphisms and PCOS. In a case-control sample using 60 PCOS patients and 92 healthy controls all being unrelated Han Chinese from Shanghai the haplotypes covering components Thr307Ala and Asn680Ser were studied. These authors observed a significant association of both polymorphisms and PCOS (24).

Ten case-control studies were included in the first meta-analysis addressing the relationship between the commonest FSHR polymorphisms and PCOS. This meta-analysis showed no consistent association between either the Thr307Ala polymorphism or the Asn680Ser polymorphism and susceptibility to PCOS. Stratified analysis of ethnicities also showed no association. The authors of this meta-analysis suggested that the FSHR polymorphisms were not associated with an increased risk of PCOS (25). In a second meta-analysis data from 11 studies were analyzed. The pooled odds ratio and 95% confidence interval were calculated using fixed- or random-effect model based on heterogeneity test in 5 genotype models analyses. This analysis showed that the Asn680Ser polymorphism was significantly associated with the reduced susceptibility to PCOS in a dominant model as well as in recessive, homozygote comparison, and allele contrast models. All Odds ratios were similar reducing the chance of having PCOS with about 20%. However, no significant associations were found between Thr307Ala and PCOS (26).

In a Korean study recruiting 235 PCOS patients and 128 control subjects, all within their reproductive age years, from Seoul. The FSHR polymorphisms Ser680Asn and Ala307Thr genotype frequencies were measured. They found that the Ser680Asn of FSHR was significantly more often associated with PCOS. In contrast the Ala307Thr variant was not at all associated with PCOS. However, their haplotype analysis revealed that both the Ser680Asn and Ala307Thr polymorphisms did not constitute a risk factor for PCOS (27). In another study genotyping was performed in 377 women with PCOS and 388 age-matched controls all from South Korean origin. Findings of this study suggest a significant association between FSHR gene polymorphisms Thr307Ala and the Asn680Ser allele frequencies and PCOS. The homozygote variant genotype results in significantly higher risk of PCOS (28). In a population of 522 Japanese women, the overall frequency of Asn/Asn, Asn/Ser, and Ser/Ser was 41.0, 46.9, and 12.1%, respectively. In polycystic ovary patients, the Asn/Ser population was significantly larger when compared with the spontaneously ovulating group (29). In a Pakistani study genomic results from 96 women with PCOS were compared with those from 96 healthy controls from the Punjab region in Pakistan. This study provides evidence of statistically significant associations between susceptibility to PCOS in Pakistani women and the gene polymorphisms in FSHR, the LHHCG-Receptor, LH-β chain and both ER-α and ER-β receptor genes (30). In another study in 386 Thai women with chronic anovulation either without (121 women) or with PCOS (133 women) using 132 known ovulatory fertile women as controls no association between the FSHR gene polymorphism at codons 307 and 680 and PCOS was found (31).

The first published European study assessed the distribution of the two most common FSHR polymorphisms in 148 normogonadotropic anovulatory infertile women and in 30 normo-ovulatory controls. Normogonadotropic anovulatory infertile patients have a different FSH receptor genotype than do normo-ovulatory controls (32). In another study in Dutch women with PCOS FSHR variants were strongly associated with the severity of clinical features of PCOS. The findings in a discovery cohort of 240 women with PCOS were replicated in another independent sample of 185 women suffering from PCOS and showed that the Ser/Ser genotype was associated with higher endogenous levels of FSH, LH and testosterone (19, 20).

In a study in Turkish adolescent girls the possible association between SNPs of the FSHR was studied. Samples from 44 adolescent girls with PCOS and 50 healthy controls were compared. Polymorphic loci on the FSHR (A307T, N680S) genes did not reveal any significant differences between cases and controls. These data do not support an association between SNPs of the FSHR and the susceptibility to PCOS in Turkish adolescent girls (33). Another study in Caucasians included 294 premenopausal Caucasian patients with PCOS, and 78 women with regular menorrhea and without hirsutism. In this study in Polish women no differences in genotype and allele frequencies of the Ser680Asn and Ala307Thr polymorphisms between the case and the control groups. In addition, the two FSHR variants in exon 10 did not increase the risk for PCOS in Polish women (34).

To assess the cross-ethnic effect a meta-analysis of the Dutch data (703 Dutch PCOS patients and 2,164 Dutch controls) combined with results of previously published studies in PCOS patients from China (*n* = 2,254) and the United States (*n* = 2,618) has been performed. Overall, this study observed for 12 of 17 genetic variants mapping to the Chinese PCOS loci similar effect size and identical direction in PCOS patients from Northern European ancestry, indicating a common genetic risk profile for PCOS across populations. In this study two previously identified GWAS FSHR polymorphisms, i.e., rs2268361 and rs2349415 were significantly associated with PCOS (15). Another study trying to replicate the Chinese GWAS findings in Caucasians included 905 women with PCOS and 956 control women. The strongest evidence for association mapping to FSHR was observed with rs1922476. Furthermore, markers with the FSHR gene region were associated with FSH levels in women with PCOS. Fine mapping of the chromosome 2p16.3 Chinese PCOS susceptibility locus in a European ancestry cohort provides evidence for association with two independent loci and PCOS. The gene products of the FSHR gene are therefore likely to be important in the etiology of PCOS, regardless of ethnicity (35). An American study tried to replicate the FSHR variants from the Chinese GWA studies in case-control examination in a discovery cohort of 485 women with PCOS and in 407 controls from Boston. Replication was performed in women from 884 PCOS cases and 311 controls from Greece and in an additional cohort from Boston constituting 350 cases and 1,258 controls. One variant, rs2268361-T, in the intron of FSHR was associated with PCOS and lower FSH levels (36). In an effort to replicate the hits for the Chinese GWA studies 845 European subjects with PCOS and 845 controls were recruited into this study. Variants in DENND1A, THADA, the FSHR, and the INS-Receptor were associated with PCOS in Europeans. The genetic risk score, generated for each subject based on the total number of risk alleles, was associated with the diagnosis of PCOS and remained associated even after exclusion of the four variants individually associated with PCOS (37). Finally, in an Arab case-control study, involving 203 women with PCOS, and 211 age- and ethnically-matched control women. Significantly lower frequencies of a heterozygous FSHR variant (rs11692782) genotype carriers were observed between women with PCOS and controls (38). In summary most studies in Chinese and Thai women did not substantiate an increased susceptibility for PCOS based on FSHR polymorphisms. In contrast Studies in Korean and Pakistani women did find an increased susceptibility for PCOS based SNP's in the FSHR. Results from meta-analysis are contradictory whereas most studies in women from Caucasian descent revealed a clear-cut increased susceptibility for PCOS based on the FSHR genotype (Table 1).

**Table 1:** Showing the effect different single nucleotide polymorphisms have on either the prevalence of PCOS or on different phenotypical features of the syndrome.

**SNP**	**Location**	**Risk for PCOS**	**Effect**	**Ethnicity**	**References**
**FOLLICLE-STIMULATING HORMONE RECEPTOR (FSHR)**					
FSHR	rs6165 rs6166	Similar Similar	Not reported Not reported	Chinese	(21)
FSHR	rs6165 rs6166	Similar Similar	No effect Higher FSH and lower E_2_	Chinese	(22)
FSHR	rs6165 rs6166	Similar Similar	Higher FSH and Prl Higher FSH and Prl	Chinese	(23)
FSHR	rs6165 rs6166	Similar Increased	Not reported Not reported	Korean	(27)
FSHR	rs6165 rs6166	Increased Increased	No effects on FSH, ovarian or metabolic markers No effects on FSH, ovarian or metabolic markers	Korean	(28)
FSHR	rs6165 rs6166	Similar Increased	No effect Reduced FSH sensitivity Lower E_2_ levels at OPU	Japanese	(29)
FSHR	rs6165 rs6166	Similar Increased	Not reported Not reported	Pakistani	(30)
FSHR	rs6165 rs6166	Similar Similar	Not reported Not reported	Thai	(31)
FSHR	rs6165 rs6166	Similar Increased	No effect Higher FSH levels	European	(32)
FSHR	rs6165 rs6166	Similar Increased	No effect!!!!! Higher FSH levels	European	(19)
FSHR	rs6165 rs6166	Similar Increased	No effect Higher FSH and LH and Testosterone levels	European	(39)
FSHR	rs6165 rs6166	Similar Increased	No effect Higher FSH levels	European	(20)
FSHR	rs6165 rs6166	Similar Similar	Not Reported!!!!! Not Reported	European	(34)
FSHR	rs6165 rs6166	Similar Similar	No effect No effect	Turkish	(33)
FSHR	rs7562215 rs10495960 rs2956355 rs7562879 rs13405728	Increased Increased Increased Increased Increased	Not reported Not reported Not reported Not reported Not reported	Caucasian	(35)
FSHR	rs2268361	Increased	Lower FSH serum levels	Caucasian	(36)
FSHR	rs11692782	Decreased	Not reported	Arab	(38)
FSHR	rs2268361 rs2349415	Not reported Not reported	More PCOM Different levels of FSH, E_2_ and SHBG Different levels of SHBG	Chinese	(40)
FSHR	rs6165 rs6166	Similar Similar	No differences in OI outcome No differences in OI outcome	Caucasian	(41)
FSHR	rs6165 rs6166	Similar Similar	No differences in OI outcome No differences in OI outcome	Caucasian	(42)
FSHR	rs6165 rs6166	Increased Similar	Higher ovarian responsiveness in OI Similar ovarian responsiveness in OI	Caucasian	(43)
FSHR	rs6165 rs6166	Similar Similar	Not reported Not reported	Chinese (Meta-analysis)	(25)
FSHR	rs6165 rs6166	Similar Decreased	Not reported Not reported	Chinese (Meta-analysis)	(26)
FSHR	rs2268361 and rs2349415	Increased Increased	Not reported Not reported	Caucasians and Chinese (Meta-analysis)	(15)
**FOLLICLE-STIMULATING HORMONE ß CHAIN POLYMORPHISMS (FSHß)**					
FSHß	T to C transition at codon 76 (AccI)	Increased in Obese PCOS women	Higher FSH levels in AccI cariers and No deifferences in E_2_ and LH values	Chinese	(44)
FSHß	rs11031010	Increased	Higher LH serum levels	Chinese	(10)
**LUTEINIZING HORMONE- AND HUMAN CHORIOGONADOTROPHIN-RECEPTOR (LHCG) POLYMORPHISMS**					
LHCGR	rs7371084 rs4953616	Increased Increased	Not reported	Arab	(38)
LHCGR	rs13405728	Increased	higher serum level of testosterone, triglycerides and low-density lipoproteins	Chinese	(45)
LHCGR	rs13405728 rs7562879	Similar Increased	Not reported Not reported	Caucasians	(35)

### FSHR Polymorphisms and Clinical Features of PCOS

One study aimed to investigate whether the PCOS related SNPs in the FSHR gene are associated with PCOM. The 447 unrelated Han Chinese PCOS from south China were grouped into PCOM (*n* = 384) and non-PCOM (*n* = 63) women. Significant differences were found in the allele distributions of the GG genotype of rs2268361 between the PCOM and non-PCOM groups while no significant differences were observed in the allele distributions of the GG genotype of rs2349415. When rs2268361 was considered, there were statistically significant differences of serum FSH, estradiol, and sex hormone binding globulin between genotypes in the PCOM group. In case of the rs2349415 SNP, only serum sex hormone binding globulin was statistically different between genotypes in the PCOM group (40). Similar findings were reported another study showing that the Ser/Ser genotype had significantly higher basal level of serum FSH was observed as compared with that in the Asn/Ser group (29). A study trying to replicate the GWAS findings of the first two Chinese studies in Caucasians found a similar relationship between FSHR polymorphisms and lower FSH levels (36). Several Dutch studies found similar relationships between the Ser/Ser FSHR variant and higher basal endogenous FSH serum concentrations (19, 20, 39). In one study it was also reported that the Ser/Ser variant was also associated with increased serum concentrations of LH and testosterone (39). There is only limited information available regarding the relationship between FSHR SNP's and the phenotype of PCOS. Some SNP's are associated with the PCOM whereas most data are showing a more strict relationship between higher basal FSH serum levels and the least sensitive FSHR (Table 1).

## FSH Receptor Polymorphisms and Treatment Outcome in PCOS

In order to determine whether an allelic variant of the FSHR gene affects fertility parameters in women with PCOS a UK group of investigators studied 93 women with PCOS and compared those with 51 healthy controls. The allelic variant Thr307/Ser680 was found to be similarly prevalent in both study groups and had no phenotype in terms of fertility parameters in women with PCOS (41). In a study comparing 58 women with PCOS and 80 healthy ethnically matched female controls there was no evidence that the Asn680Ser FSHR genotypes were neither associated with PCOS nor with the response to clomiphene citrate (42).

In contrast in a population of 522 Japanese women the NS genotype was significantly more prominent in women with PCOS. In women with the SS genotype significantly higher basal serum levels of FSH were observed as compared with the NS group suggesting a lower sensitivity of this particular SNP. Indeed, higher doses of the exogenous FSH was required to achieve ovulation induction in the SS group. Moreover, after hCG administration, estradiol levels at the time of ovum pick-up were significantly lower in the SS group as compared to the other allelic variants. In case the two receptor variants were over expressed in 293T cell line no differences could be found in either levels of FSH-stimulated cAMP production, PI turnover or ligand-binding affinity. These results suggest some FSHR variants might have clinical implications (29). Similar results have been reported in a large Dutch case control cohort of women with PCOS and healthy controls the Ser/Ser FSHR polymorphism was associated with some clinical features such as FSH and LH serum concentrations. Indeed, the Ser/Ser FSHR polymorphism seemed to be less sensitive to endogenous FSH thereby leading to higher serum concentrations of FSH. Surprisingly this variant was also associated with higher serum LH and testosterone levels (32, 39). In the same study the Dutch group revealed an association with clomiphene citrate resistance during ovulation induction treatment. A pooled analysis showed an 89% higher chance of being CRA in homozygous carriers of the Ser/Ser FSHR variant. Similarly, a lower chance of ongoing pregnancy [hazard ratio 0.51 (95% confidence interval 0.27–0.98)] was observed among these patients during clomiphene citrate treatment in two independent prospective cohorts (39). These data may be used to design a treatment algorithm that is more efficacious and better tailored to the individual patient (20).

Similar results were obtained from another Dutch group retrospectively studying a cohort of 193 patients all diagnosed with PCOS according to Rotterdam criteria and treated with ovulation induction. Significantly more patients with Ser/Ser-polymorphism were resistant to CC compared with the Asn/Ser and Asn/Asn genotypes with an odds ratio for ovulation of 0.44. Patients with higher FSH levels, higher age and lower BMI were significantly more likely to ovulate in univariate analysis. In a multivariate logistic regression model, corrected for age, BMI, mean ovarian, volume, hyperandrogenism, and amenorrhea, only the FSHR genotype and basal FSH serum levels were predictive for ovulation (19). An Italian study compared 40 women with PCOS undergoing *in-vitro* fertilization (IVF) with 66 normo-ovulatory women. That study showed that the heterozygote FSH-R polymorphism Ala307Thr was significantly more frequent in women with PCOS compared to the normo-ovulatory subjects. Moreover, the Ala307Thr SNP was more frequently associated with a higher ovarian responsiveness to exogenous FSH (43). Although smaller studies in Caucasian women did not reveal a clear-cut relationship between FSHR polymorphisms and treatment outcome the larger studies did reveal an increase in clomiphene citrate resistance and the least sensitive FSHR (Ser680Ser variant) (Table 1).

## Other Genetic Variants Gonadotrophic Regulation that Might be Associated With PCOS

### FSH-β Gene Polymorphisms

In a cohort of 135 patients with PCOS and 105 normal control subjects no missense mutations were found in the functional units of the FSH-β gene in patients with PCOS. However, this study identified a thymine-cytosine substitution in exon 3 (codon 76, TAT to TAC) that led to creation of a so called AccI digestion site. The distribution pattern of this AccI polymorphism in the patients was significantly different from that in the control group since homozygous carriers were more often affected by PCOS. Within the PCOS patient group homozygosity for Accl was also associated with obesity. The latter finding correlated with significantly higher androgen levels in the obese patients. Hence the AccI polymorphism in FSHβ gene may be associated with PCOS in some women, especially those with obesity (44). A meta-analysis of Chinese GWAS data showed that the allele frequency difference of a SNP in the FSH-β gene (rs11031010) between PCOS and controls was genome-wide significant. PCOS women with AA and AC genotypes had a significantly higher LH serum levels compared to women carrying the CC genotype. Hence, variants in FSH-β gene are associated with PCOS and LH levels in Han Chinese women. The FSH-β gene is thus likely to play an important role in the etiology of PCOS, regardless of ethnicity (10) (Table 1).

### LHCG-Receptor Polymorphisms

In a retrospective case-control study, involving 203 women with PCOS, and 211 age- and ethnically-matched control women LHCGR genotyping was done by allelic exclusion method. Significantly lower frequencies of heterozygous LHCGR rs7371084 genotype carriers were seen between women with PCOS vs. controls. Furthermore, an increased frequency of heterozygous homozygous LHCGR rs4953616 genotype carriers was detected between women with PCOS compared to control women. The authors of this study observed a significant increase of LHCG-Receptor variants (rs7371084, rs4953616) SNP's in women with PCOS (38). In a case control study in Hui ethnic women from China 51 patients with PCOS and 99 healthy women were involved. The frequencies of the genotype and allele frequency of rs13405728 in LHCG-Receptor gene were significantly different between the PCOS and the control women. Moreover, PCOS cases with TT genotype of the variant rs13405728 had higher serum level of total testosterone, triglycerides, and low-density lipoproteins (LDL) than those with the CC and CT genotypes. The authors concluded that the SNP rs13405728 in the LHCG-Receptor gene was associated with PCOS and some of its clinical features (45). An attempt to replicate the Chinese SNP's in an American cohort revealed that the LHCG-Receptor variant (rs13405728) was not informative in a white Americans from European descent. However, these authors identified and genotyped three markers (rs35960650, rs2956355, and rs7562879) within 5 kb of rs13405728. Of these, rs7562879 was nominally associated with PCOS. The gene products of the LHCRG-Receptor gene are therefore likely to be important in the etiology of PCOS, regardless of ethnicity (35).

In Indian study involving 204 women with PCOS and 204 healthy, sex-, and age-matched controls. This study demonstrated an association between LHCGR (rs2293275) polymorphism and PCOS. Moreover, a significant association of the GG allele with body-mass index, waist to hip ratio, insulin resistance, LH, and LH/FSH ratio was demonstrated in PCOS when compared with controls. Indeed this study also suggests that LHCG-Receptor polymorphism are associated with PCOS (46) (Table 1).

## Summary

Data from Chinese studies regarding the relationship between FSHR polymorphisms and PCOS susceptibility are conflicting. Although the majority of data do not substantiate such a relationship (21–23) there is one report suggesting that FSHR might play a role in genetic susceptibility to PCOS (24). Two different meta-analysis revealed also contradictory results. The first one suggested that the FSHR polymorphisms were not associated with an increased risk of PCOS (25) whereas the last one did reveal a decreased susceptibility to PCOS for Thr307Ala variant carriers (26). Several Korean (27, 28) as well as Japanese (29) and Pakistani studies (30) do substantiate the FSHR variants as a susceptibility locus for PCOS. However, a study in Thai women did not reveal such a relationship (31). It seems that data from South-East Asia are different from those generated in Chinese populations. In European women data are similarly conflicting. Some Dutch studies revealed a straight on forward association between FSHR gene polymorphisms and PCOS (19, 20, 39) whereas others did not substantiate such susceptibility (17, 33, 34). Arab women carrying certain FSHR polymorphisms seem to be more often suffering from PCOS too (38). The most convincing evidence comes from larger replication studies and a cross ethnic meta-analysis that revealed a strong relationship between FSHR variants and PCOS susceptibility (15, 35–37).

In conclusion along with these more convincing data observations underpinning the association of FSHR polymorphisms with clinical features indicating differences in sensitivity of the different receptor genotypes. These receptor variants might generate several phenotypes with differences in basal serum FSH, LH, and testosterone concentrations. Some of the data are also pointing in a direction that at least some of these genetic variants have clinical implications in that they determine individual sensitivity for exogenous FSH (19, 29, 32, 39, 42, 43). The observation that treatment outcome is also partially determined by FSHR polymorphisms is another clue that genetic variants might play a role in the pathophysiology of PCOS (19, 20, 29, 39, 42, 43).

In conclusion SNP's in the FSHR gene causing genetic variants in the FSHR on the one hand do determine the susceptibility for PCOS and on the other had do also affect the sensitivity of the receptor for exogenous FSH during ovulation induction therapy. There is also limited evidence showing that SNP's in the LHCG-Receptor as well as those in FSH-β gene might also determine a women's susceptibility for PCOS.

## Author Contributions

JL delivered a substantial contribution to the conception of the work and the interpretation of data in the literature for the work. He drafted the work and agrees to be accountable for all aspects of the work in ensuring that questions related to the accuracy or integrity of any part of the work are appropriately investigated and resolved.

### Conflict of Interest Statement

The author declares that the research was conducted in the absence of any commercial or financial relationships that could be construed as a potential conflict of interest.
